# Umbilical Cord Stump Infections in Central Uganda: Incidence, Bacteriological Profile, and Risk Factors

**DOI:** 10.3390/ijerph192316055

**Published:** 2022-11-30

**Authors:** Josephine Tumuhamye, Halvor Sommerfelt, James K. Tumwine, David Mukunya, Grace Ndeezi, Olive Namugga, Freddie Bwanga, Hans Steinsland, Victoria Nankabirwa

**Affiliations:** 1 Centre for Intervention Science for Maternal and Child Health, Centre for International Health, Department of Global Public Health and Primary Care, University of Bergen, 5020 Bergen, Norway; 2 Cluster for Global Health, Division for Health Services, Norwegian Institute of Public Health, 0430 Oslo, Norway; 3 Department of Paediatric and Child Health, Makerere University, Kampala P.O. Box 7062, Uganda,; 4 School of Medicine, Kabale University, Kabale P.O. Box 317, Uganda; 5 Department of Community and Public Health, Busitema University, Mbale P.O. Box 236, Uganda; 6 Department of Immunology and Molecular Biology, Makerere University, Kampala P.O. Box 7062, Uganda; 7 Department of Biomedicine, University of Bergen, 5020 Bergen, Norway; 8 Department of Epidemiology, Biostatics School of Public Health, Makerere University, Kampala P.O. Box 7062, Uganda

**Keywords:** omphalitis, umbilical cord stump infections, neonates, newborns, incidence rate, incidence proportion, antimicrobial-resistance

## Abstract

Umbilical cord stump infection (omphalitis) is a risk factor for neonatal sepsis and death. We assessed the incidence of omphalitis, described the bacteriological and antibiotic-resistance profile of potentially pathogenic bacteria isolated from the umbilical cord stump of omphalitis cases, and evaluated whether bacteria present in the birth canal during birth predicted omphalitis. We enrolled 769 neonates at birth at three primary healthcare facilities and followed them for 28 days with scheduled visits on days 3, 7, 14, and 28. Cox regression models were used to estimate the rates of omphalitis associated with potential risk factors. Sixty-five (8.5%) neonates developed omphalitis, with an estimated incidence of 0.095 cases per 28 child-days (95% CI 0.073, 0.12). Potentially pathogenic bacteria were isolated from the cord stump area of 41 (63.1%) of the 65 neonates with omphalitis, and the most commonly isolated species were *Escherichia coli* (*n* = 18), *Klebsiella pneumoniae* (*n* = 10), *Citrobacter freundii* (*n* = 5), and *Enterobacter* spp. (*n* = 4). The *Enterobacteriaceace* isolates were resistant to gentamicin (10.5%, 4/38), ampicillin (86.8%, 33/38), and ceftriaxone (13.2%, 5/38). Delayed initiation of breastfeeding was associated with an increased risk of omphalitis (aHR 3.1; 95% CI 1.3, 7.3); however, vaginal colonization with potentially pathogenic bacteria did not predict omphalitis.

## 1. Introduction

Globally, 5.1 million children die before five years of age, and of these deaths, 2.4 million occur during the first month of life [1]. In Uganda, it has been estimated that out of every 1000 live births, almost 307 die within the first 28 days of life [2], i.e., as neonates. Infections are the leading cause and account for approximately one-third of all neonatal deaths in sub-Saharan Africa [3]. Omphalitis is clinically characterized by the presence of redness, swelling, and/or pus at the umbilical cord stump [4]. The stump can act as a portal of entry for invasive pathogenic bacteria into the bloodstream [5]. Furthermore, it contains necrotic tissue, which provides a suitable medium for bacterial growth. The mother’s birth canal is one of the most important sources of these bacteria, where the umbilical cord is contaminated during birth [6]. Other known maternal risk factors associated with omphalitis include intrapartum infections such as chorioamnionitis, a prolonged rapture of membranes, and home delivery; neonatal risk factors include very low birth weight and improper cord care, such as the application of home remedies to speed up cord separation, which often facilitates bacterial contamination and increases the risk of infection [7]. Potentially pathogenic bacteria that are commonly found colonizing the umbilical cord include *Staphylococcus aureus*, group A *streptococci*, group B *streptococci*, and gram-negative bacteria such as *Escherichia coli*, *Klebsiella* species, *Enterobacter* species, and *Pseudomonas* species.

Omphalitis remains an important risk factor for severe illness and death among neonates in developing countries [4,8,9]. Four large community-based trials in developing countries indicate that the incidence risk of moderate to severe omphalitis during the neonatal period may range widely, from 1% to 7.6% [10,11,12,13]. Although neonatal infections are a major problem in developing countries, data on the incidence, bacterial etiology, antimicrobial resistance patterns, and risk factors associated with the development of neonatal omphalitis are scarce. Previous studies focus mainly on the incidence of omphalitis [10,11,12,14,15,16] but few report on the risk factors, bacterial etiology, and antimicrobial resistance patterns [7,15,16]. We sought to estimate its incidence among HIV-unexposed babies born at three primary healthcare facilities in Uganda. We also describe the bacteriological profile of the cord stump of the omphalitis cases. Finally, we estimate the association between vaginal colonization and potentially pathogenic bacteria of women in labor and omphalitis amongst their neonates and sought to describe other factors that may be associated with neonatal omphalitis.

## 2. Materials and Methods

### 2.1. Study Design and Setting

We conducted a prospective cohort study between July 2016 and July 2018 at three primary healthcare facilities in Central Uganda: Mukono general hospital (formerly Mukono Health Centre IV), Kawaala Health Centre III, and Kitebi Health Centre III. The two health centers are located in urban slum areas in Kampala district, the capital city of Uganda. Both these health facilities mostly conducted normal deliveries during the study period, while Mukono general hospital, which is located within Mukono district about 20 km away from Kampala, also conducted more complicated deliveries, including caesarian sections. This study was nested within a randomized controlled trial assessing the effectiveness of a single application of 4% chlorhexidine solution on the umbilical cord stump for the prevention of omphalitis and severe illness in neonates whose mothers did not have HIV infection [17]. We enrolled participants from the standard care (control) arm of this clinical trial, where the cord stump was left dry. The present study includes data from two previous studies that characterized potentially pathogenic bacteria isolated from vaginal swabs from the mothers during labor [18,19].

### 2.2. Recruitment, Enrollment, and Follow-Up

During recruitment, a study midwife obtained permission verbally from women in labor to collect vaginal swabs. After giving birth, a study nurse informed the women about the chlorhexidine trial [17] and obtained informed consent for enrolment into the trial and the current cohort study. The exclusion criteria were birth weight less than 1.5 kg, omphalitis at birth, severe congenital anomaly, and severe illness requiring immediate hospitalization. In the current study, we aimed to include the 800 newborn babies enrolled in the dry cord care (control) arm of the trial when its sample size was planned to be 1600 [17].

After enrollment, the neonate was scheduled for follow-up, on day 3, day 7, day 14, and day 28. During these interviews, a study nurse examined the baby for various health indicators, including signs of omphalitis, and asked the mother about past symptoms and illnesses. The scheduled visits had specified window periods around them, i.e., day 2 to day 4 for the day 3 visit, day 5 to day 9 for the day 7 visit, and day 10 to day 18 for the day 14 visit. The window period for the day 28 visit was from day 22 to day 40. If a mother did not turn up for an interview, reminder phone calls were made, and if this failed then study staff made home visits to speak directly with the mother and bring her and her baby to the study clinic. If no interviews could be conducted within the specified time window, the participant was considered to be lost to follow-up for the scheduled visit.

We set out to enroll 800 neonates in the control arm of the trial from mid-2016 to mid-2018, i.e., half of its initial sample size of 1600. This would yield a high (1.0% to 2.5%) absolute precision (half-width of the 95% confidence interval) for omphalitis risk in the range of 2% to 15%.

### 2.3. Data Collection, Management, and Quality Control

As described earlier [19], we used electronic questionnaires generated using the Open Data Kit (ODK) software [20] on mobile phones to capture our data. The pre-coded digital questionnaires had inbuilt checks to prevent progression if erroneous data were entered. The completed questionnaires were saved on mobile phones and uploaded onto a server at the end of each day. During enrollment, we collected baseline data, including birth weight, baby sex, breastfeeding initiation (early: within one hour after birth, after which we characterized it as delayed), and the mother’s age, education, and socioeconomic status as well as mode of delivery (vaginal or caesarean section).

Omphalitis was in the current study defined by the presence of pus on the umbilical cord stump [17]. Before study began, we trained the study nurses in the study procedures, including the use of structured electronic questionnaires and yearly refresher sessions during the study to ensure data quality and completeness.

### 2.4. Specimen Collection

We collected umbilical cord stump swabs for bacterial culture from babies with omphalitis during the follow-up visits scheduled for days 3, 7, 14, and 28 after birth. Using Regular Rayon sterile swabs pre-packed with Amies agar gel transport medium without charcoal (Copan Diagnostics Inc., Murrieta, CA, USA), a trained midwife moved a swab gently around the cord stump after which she placed it into a specimen tube containing the transport medium and kept it refrigerated for up to 8 h before the bacteriological analyses were undertaken.

### 2.5. Bacterial Isolation and Identification

The bacterial isolation and identification were performed as described previously [18,19]. Briefly, primary inoculation of the umbilical swab was performed on 5% sheep blood agar and MacConkey agar (both from BioLab Zrt., Budapest, Hungary), followed by aerobic incubation between 35 °C to 37 °C for 18 to 24 h for MacConkey plates and blood agar plates up to 72 h to allow growth of slow-growing bacteria. In this cohort study, we considered the following species to be potentially pathogenic: *Staphylococcus aureus*, *Escherichia coli*, *Klebsiella pneumoniae*, group A *Streptococcus* (GAS), group B *Streptococcus* (GBS), *Enterococcus* spp., *Pseudomonas* spp., *Enterobacter* spp., *Citrobacter* spp., *Proteus* spp., and *Acinetobacter* spp., since they are known to cause infections in neonates. We considered all other species of bacteria that were isolated in this study as being commensals since they rarely are associated with neonatal infections, and these isolates were therefore excluded from further analyses. These included isolates of *Micrococcus* spp., *Corynebacterium* spp., *Lactobacillus* spp., *Bacillus* spp., *Bukolderia* spp., *Serratia* spp., and coagulase-negative Staphylococci.

### 2.6. Antimicrobial Susceptibility Testing

Antimicrobial drug susceptibility testing of the bacterial isolates was performed on Mueller-Hinton agar plates by using the disk diffusion method as described earlier [18] based on the Clinical Laboratory Standard Institute (CLSI) guidelines [21]. All antibiotic disks were produced by and purchased from BioLab Zrt.

### 2.7. Statistical Analysis

Data were analyzed by using Stata version 17.0 (StataCorp, College Station, TX, USA). We summarized categorical variables as proportions and continuous variables as means and compared them by using Student’s t-tests. We defined the neonatal incidence risk of omphalitis as the proportion of newborns who had at least one episode of omphalitis during their first 28 days of life. When calculating the incidence risk for each specific follow-up period (from birth to day 3, day 4 to 7, day 8 to 14, and day 15 to 28), we based the calculations on the mid-period population size. For example, the mid-period population for the birth to day 3 period was calculated as the study population count at enrollment plus the number of neonates who completed the day 3 examination, divided by two.

We calculated person-time under observation by summing up the time each neonate contributed up to 28 days of life or until they were censored (developed omphalitis, died, or were lost to follow-up). Time to the outcome was calculated as the age of the neonate, in days, at the time they were first diagnosed with omphalitis. Neonates who did not develop omphalitis contributed person-time up until their last registered visit to the clinic. The neonates did not contribute person-time during missed visits because of these neonates, we based the diagnosis of omphalitis on information obtained from the mothers during the first subsequent visit. According to their caretaker, none of the neonates who missed a scheduled visit experienced omphalitis during that period. We defined incidence rate of omphalitis as the ratio of the number of new omphalitis cases divided by the total person-time under observation. Incidence rate was converted to risk by using the formula: incidence rate × person-time under observation [22].

Kaplan-Meier curves were generated using the sts graph code in Stata. To estimate the association between vaginal colonization during birth and potentially pathogenic bacteria and subsequent neonatal omphalitis, we fitted a Cox proportional hazard regression model to estimate the hazard ratio (HR). The final model included vaginal colonization as the main exposure, and level of education, maternal age, socio-economic status, baby sex, birthweight, and delayed initiation of breastfeeding as potential cofounders. We evaluated the covariates for collinearity, defined as presence of a variance inflation factor greater than 10 [23].

## 3. Results

### 3.1. Participant Characteristics

**Figure 1:**
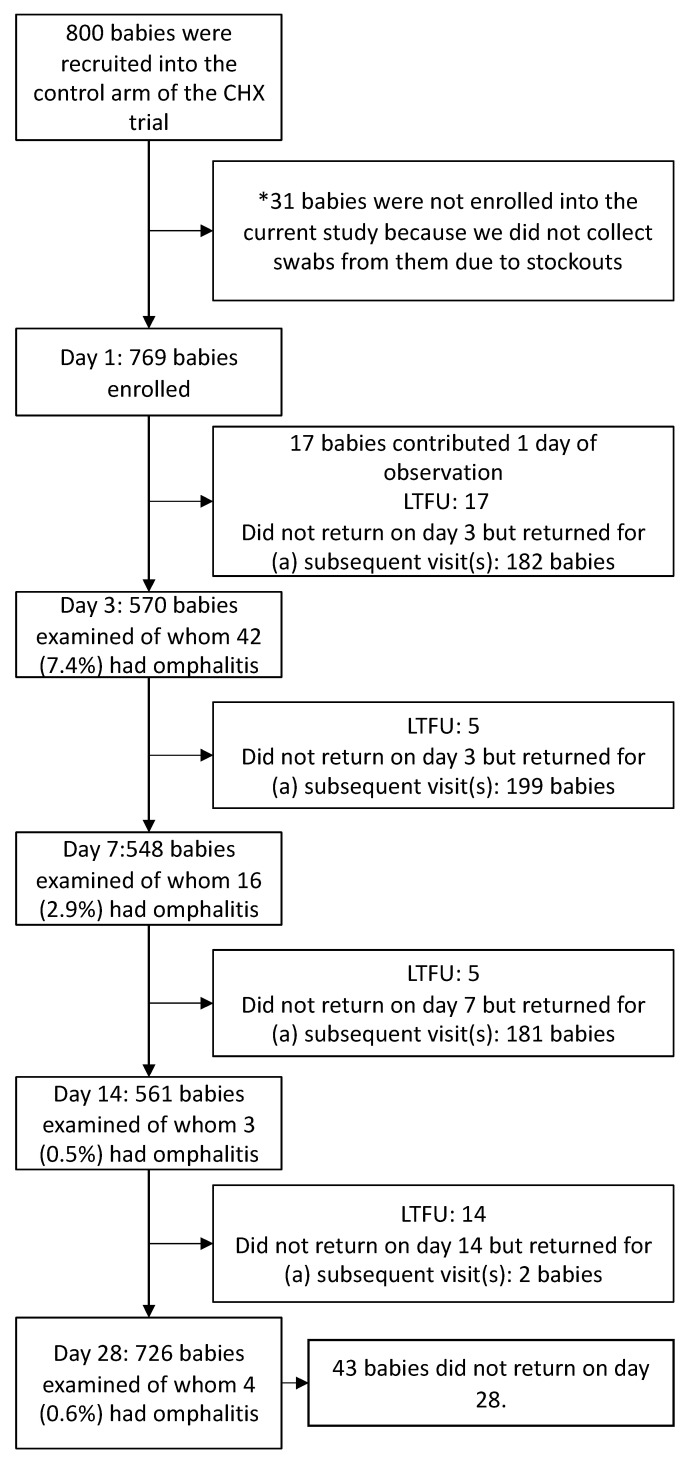
Flow chart of participants included in the study. MLFTU: Lost to follow-up; babies that never returned for the subsequent scheduled visits. * The missing data resulted from stockouts of swabs that were spread out during the course of the study.

Of the 800 neonates in the control arm of the chlorhexidine trial, we enrolled 769 (96.1%), the remaining 31 could not be examined because swabs were occasionally out of stock (Figure 1). The participants’ mean birth weight was 3.2 kg (standard deviation 0.4). Half of them (52.7%) were males (405/769) and only three were delivered by caesarian section, the rest vaginally. Most (95.7%) study babies (736/769) initiated breastfeeding within the first hour and 13.8% (106/769) of neonates had home remedy substances applied to the umbilical cord stump. Follow-up proportions were 570/769 (74.2%) on the visits scheduled for day 3, 548/769 (71.3%) for day 7, 561/769 (73.0%) for day 14, and 726/769 (94.4%) for day 28. The mothers of the neonates had a mean age of 25 years (SD 4.8) at birth. Two-thirds were vaginally colonized with at least one potentially pathogenic bacterium during labor (Table 1).

### 3.2. Incidence of Omphalitis

Sixty-five (8.5%) of the 769 babies developed omphalitis during follow-up and the majority (58 or 89.2%) of the 65 cases occurred in the first week of life. The risk of omphalitis was 6.3% by day 3 (42/670; 95% CI 4.0%, 7.3%), 2.9% from day 4 to day 7 (16/559; 95% CI 1.3%, 3.5%) and 1.1% between day 8 and day 28 (7/644; 95% CI 0.40%, 2.0%). The majority (89.2%) of the 65 cases occurred in the first week of life. Overall, the neonatal incidence rate of omphalitis was 0.095 cases per 28 child-days (95% CI 0.073, 0.12), translating to a neonatal cumulative incidence, i.e., risk, of 9.5% (95% CI 7.3%, 12.1%). All cases of omphalitis included in these calculations were new, i.e., incident cases.

### 3.3. Bacteriological Profile of Omphalitis

Among the 65 neonates who developed omphalitis, we isolated at least one species of potentially pathogenic bacteria from 41 (63.1%) neonates. Of the 41 neonates, 43 potentially pathogenic bacterial species were isolated, 39 neonates were colonized with one bacterial species each while 2 neonates were colonized with two bacterial species each. All except five of the bacterial isolates were Enterobacteriaceae. The predominant potentially pathogenic bacterial species isolated from the neonates with omphalitis included E. coli 27.7% (18/65), *K. pneumoniae* 15.4% (10/65), and *C. freundii*, 7.7% (5/65). Other bacterial species included *Enterobacter* spp. 6.1% (4/65), *Acinetobacter* spp., 4.6% (3/65), *S. aureus* 3.1% (2/65), and *K. oxytoca* 1.5% (1/65). Twenty-four potentially pathogenic bacteria were isolated from neonates assessed on day 3, thirteen potentially pathogenic bacteria from cases assessed on day 7, while three potentially pathogenic bacteria were isolated from neonates observed on both days 14 and 28 of the follow-up.

### 3.4. Antimicrobial Resistance Profiles

Among the Enterobacteriaceace isolates, 86.8% (33/38) were resistant to ampicillin, 73.7% (28/38) to amoxicillin-clavulanic acid, and 60.5% (23/38) were resistant to trimethoprim-sulfamethoxazole. There was a relatively low proportion of strains that were resistant to third-generation cephalosporins, such as ceftriaxone in 13.2% (6/38), ceftazidime in 10.5% (4/38), and gentamicin in 10.5% (4/38). All organisms but one were susceptible to imipenem. One of the two *S. aureus* isolates in this study was methicillin-resistant (MRSA) and phenotypically expressed erythromycin inducible clindamycin resistance (D-test positive). Other organism-specific antibiotic resistance patterns are summarized in Table 2. We did not find extended-spectrum beta-lactamase and carbapenem-resistant gram-negative bacteria (Table 2).

### 3.5. Factors Associated with Omphalitis

**Figure 2:**
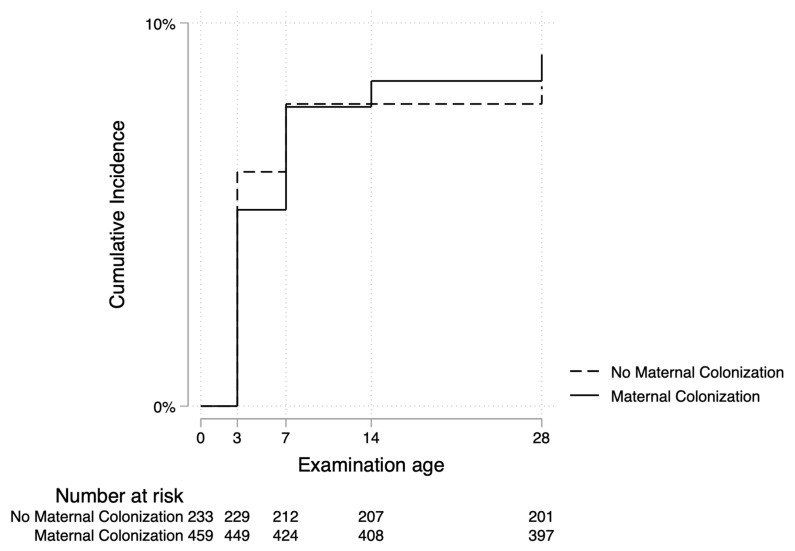
Rate of omphalitis by maternal vaginal colonization with potentially pathogenic bacteria at three study sites in central Uganda.

**Figure 3:**
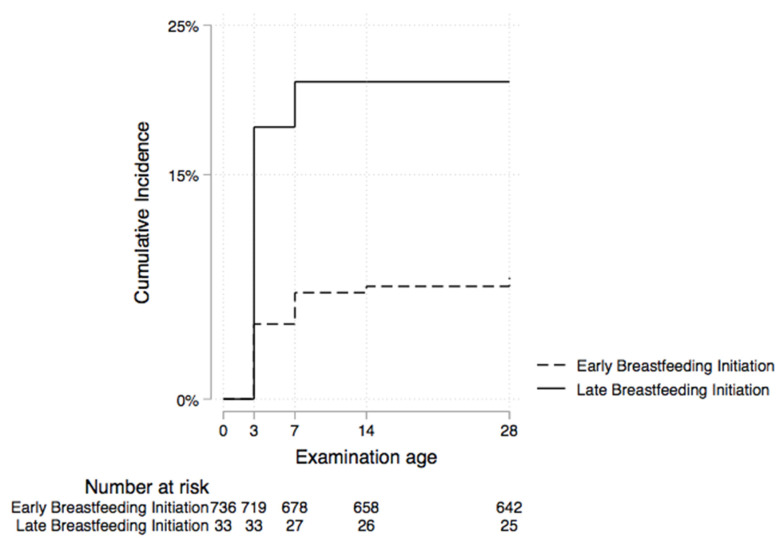
Rate of omphalitis by breastfeeding initiation status at three study sites in central Uganda.

We found that neonates of women who were vaginally colonized with potentially pathogenic bacteria were almost equally likely to get omphalitis compared to those of women who were not colonized (adjusted hazard ratio (aHR) 1.1; 95% CI 0.63, 1.9) (Figure 2 and Table 3). The analysis revealed that the hazard of omphalitis among neonates initiated late on breastmilk was approximately three times that among neonates who were initiated early (aHR 3.1; 95% CI 1.3, 7.3) (Figure 3 and Table 3). Other maternal and neonatal characteristics were not strongly associated with omphalitis (Table 3).

## 4. Discussion

In this study, we sought to estimate the incidence of omphalitis among HIV unexposed neonates born at three primary healthcare facilities in central Uganda and to describe the bacteriological profile of the cord stump of the omphalitis cases. We found that the omphalitis incidence proportion was almost 10% in the first 28 days of life. This finding is worrying given that Omphalitis is associated with neonatal sepsis in 2 out of every 100 cases [15]. Our findings suggest that interventions such as chlorhexidine that reduce the risk of omphalitis could be useful in our and similar settings [24]. This suggestion contradicts the current World Health Organization guidelines that do not recommend chlorhexidine use for babies born in health facilities [25]. However, our suggestion is in agreement with Hodgkin [26], who questions the limitation of chlorhexidine use to only home births given that the incidence of omphalitis is also high in health facility births. A cohort study conducted in Pakistan found results similar to ours (an incidence proportion of 8%) [13]; however, previous randomized control studies in Africa and Asia estimated the omphalitis incidence proportions to range from 1% to 7.6% [10,11,12,13]. The observed differences in the incidence proportions could result from varying case definitions of omphalitis across the studies. In our study, we defined omphalitis as the presence of pus while other studies (in Tanzania and Bangladesh) defined omphalitis based on redness and/or swelling, with or without pus.

We identified potentially pathogenic bacteria that could have caused omphalitis and characterized their antimicrobial susceptibility patterns. Two-thirds of neonates with omphalitis predominately grew gram-negative bacteria, similar to what was observed in two hospital-based studies in India [9,27]. However, other studies in south Asia identified gram-positive bacteria as the most likely cause of omphalitis [15,28,29]. It is not clear why we predominately isolated gram-negative bacteria rather than the expected gram-positive bacteria such as *S. aureus*. Most (87%) of the isolated gram-negative bacteria in our study exhibited resistance to the first-line antibiotics used for treating neonatal infections. We also observed resistance to third-generation cephalosporin antibiotics. These findings are consistent with those in a cohort study in Pakistan [15]. The World Health Organization recommends ampicillin and gentamicin as first-line treatment for serious neonatal infections but cloxacillin is the first-line treatment for umbilical infections because *S. aureus* is the presumed cause. However, cloxacillin as a choice of treatment becomes problematic when the umbilical cord stumps of neonates with omphalitis are primarily colonized with gram-negative bacteria. Our findings advocate for periodic and context-specific culture and sensitivity results to guide generic treatment options.

We did not observe an association between vaginal colonization of women in labor and omphalitis in this study. However, we found that neonates who were initiated late on breastmilk had an increased risk of omphalitis compared to neonates who started breastfeeding early. These findings are similar to those in a Tanzanian study [16], which found that neonates who were breastfed in the first hour of life had a lower risk of omphalitis. Initiating breastfeeding in the first hour of life has been shown to reduce neonatal morbidity and mortality, possibly through the transfer of protective antibodies in colostrum [30,31]. Our finding that neonates who were initiated late on breastmilk had an increased risk of omphalitis compared to neonates who started breastfeeding early may be due to reverse causality. The babies with sub-clinical omphalitis at birth could have failed to breastfeed in the first hour of life. Alternatively, this finding could have been a spurious result (chance) since it was not the main objective of this study and could also have resulted from the Table 2 fallacy [32]. The Table 2 fallacy suggests that the exposure association we observed could have affected the heterogeneity across levels of other covariates in our model or were confounded by other covariates in our model [32].

One of the limitations of this study was that we did not have data on the outcome among babies that were possibly taken to other health facilities spontaneously between the scheduled visits and this may have resulted in a slight underestimation of the incidence of omphalitis. We assume that the underestimation is very small because the scheduled visits were frequent (days 1, 3, 7, 14, and 28). In this cohort, only two babies died. One died on day 16 after getting omphalitis, and the second died on the 28th day (without omphalitis). Therefore, we do not think death was a substantial competing event to omphalitis in this particular cohort and therefore we did not conduct a competing risk analysis. We also studied babies that were enrolled in a randomized controlled trial and our results are subject to limitations of results from randomized controlled trials such as the possibility of reduced generalizability.

## 5. Conclusions

In conclusion, the incidence rate of omphalitis among neonates born in three primary healthcare facilities in or close to Kampala in Uganda was 0.095 cases per 28 child-days, corresponding to an estimated incidence risk of almost 10%. Predominantly gram-negative bacteria were isolated from neonates with omphalitis, and most of the bacteria exhibited resistance to commonly used antibiotics. The antimicrobial resistance of the isolated bacteria could complicate the treatment of serious neonatal infections and may continue to represent an impediment to enhancing survival until care for the cord stump is improved.

## Figures and Tables

**Table 1:** Distribution of social-demographic characteristics among study participants with and without omphalitis at the three study sites in central Uganda.

Mothers Characteristics	No Omphalitis *N* = 704 (%)	Omphalitis *N* = 65 (%)
Age		
19 years or less	94 (13.4)	11 (16.9)
20–24 years	281 (39.9)	21 (32.3)
≥25 years	329 (46.7)	33 (50.8)
Level of education		
Primary	228 (32.4)	24 (36.9)
Secondary and Tertiary	476 (67.6)	41 (63.1)
Socioeconomic status		
Quintile 1	224 (31.8)	17 (26.2)
Quintile 2	80 (11.4)	9 (13.9)
Quintile 3	124 (17.6)	9 (13.9)
Quintile 4	136 (19.3)	15 (23.1)
Quintile 5	140 (19.9)	15 (23.1)
Mode of delivery		
Spontaneous vaginal delivery	637 (90.5)	59 (90.8)
Assisted vaginal delivery	64 (9.1)	6 (9.2)
Caesarian section	3 (0.4)	0 (0)
Gravidity		
One pregnancy	212 (30.1)	18 (27.7)
Two or more pregnancies	492 (69.9)	47 (72.3)
Vaginal colonization		
No	214 (33.9)	19 (31.7)
Yes	418 (66.1)	41 (68.3)
* Missing data	72 (10.2)	5 (7.7)
Health facility		
Kawaala HC III	265 (37.6)	10 (15.4)
Kitebi HC III	248 (35.3)	28 (43.1)
Mukono general hospital	191 (27.1)	27 (41.5)
Initiation of breastfeeding		
Early (within 1 h of birth)	678 (96.3)	58 (89.2)
Late	26 (3.7)	7 (10.8)
First breast milk		
Gave it to baby	699 (99.3)	63 (96.9)
Threw it away	5 (0.7)	2 (3.1)
Birth weight		
Low	25 (3.5)	2 (3.3)
Normal	679 (96.5)	63 (96.9)
Home remedy substance applied on the cord		
No	610 (86.7)	53 (81.5)
Yes	94 (13.3)	12 (18.5)
Sex of baby		
Male	375 (53.3)	30 (46.2)
Female	329 (46.7)	35 (53.8)

* The missing data resulted from stockouts of vaginal swabs that were spread out during the course of the study.

**Table 2:** Resistance patterns of organisms isolated from neonates with omphalitis at three study sites in central Uganda.

	Number (%) of the 65 Neonates Whose Umbilical Cord Stumps Were Colonized with Potentially Pathogenic Bacteria Resistant to One or More Antiobiotics			
Antibiotic	*Escherichia coli* (*n* = 18)	*Klebsiella pneumoniae* (*n* = 10)	*Citrobacter species* (*n* = 5)	*Enterobacter species* (*n* = 4)
Ampicillin	14 (21.5)	10 (15.4)	5 (7.7)	4 (6.2)
Ampicillin-Clavulanic acid	13 (20.0)	8 (12.3)	4 (6.2)	3 (4.6)
Trimethoprim-Sulfamethoxazole	11 (16.9)	7 (10.8)	4 (6.2)	1 (1.5)
Ciprofloxacin	2 (3.1)	3 (4.6)	1 (1.5)	1 (1.5)
Chloramphenicol	2 (3.1)	3 (4.6)	2 (3.1)	0
Gentamicin	1 (1.5)	3 (4.6)	0	0
Amikacin	2 (3.1)	6 (9.2)	0	1 (1.5)
Ceftriaxone	3 (4.6)	2 (3.1)	0	0
Cefuroxime	5 (7.7)	2 (3.1)	1 (1.5)	0
Ceftazidime	1 (1.5)	2 (3.1)	0	0
Imipenem	0	2 (3.1)	0	0

**Table 3:** Factors associated with omphalitis among neonates at the three study sites in central Uganda.

Mothers Characteristics	Unadjusted HR 95% CI	Adjusted HR 95% CI *N* = 692
Vaginal colonization		
No	1	1
Yes	1.1 (0.63, 1.9)	1.1 (0.63, 1.9)
Age		
19 years or less	1.5 (0.73, 3.1)	1.4 (0.63, 3.0)
20–24 years	1	1
25 years and above	1.3 (0.76, 2.3)	1.1 (0.63, 2.0)
Level of education		
Primary	1	1
Secondary and Tertiary	0.83 (0.50, 1.4)	0.83 (0.49, 1.4)
Socioeconomic status		
Quintile 1	1	1
Quintile 2	1.4 (0.64, 3.2)	1.3 (0.56, 3.1)
Quintile 3	0.96 (0.43, 2.1)	0.91 (0.39, 2.2)
Quintile 4	1.4 (0.71, 2.8)	1.4 (0.66, 2.8)
Quintile 5	1.4 (0.69, 2.8)	1.4 (0.70, 3.0)
Birth weight		
Normal	1	1
Low	0.89 (0.22, 3.7)	1.1 (0.26, 4.4)
Initiation of breastfeeding		
Early	1	1
Late	2.8 (1.3, 6.2)	3.1 (1.3, 7.3)
Child Sex		
Male	1	1
Female	1.3 (0.80, 2.1)	1.4 (0.85, 2.4)

## Data Availability

Datasets used for this study can be obtained through reasonable request from the principal investigator of the Chlorhexidine trial, Victoria Nankabirwa.
